# Higher Negative Margin Rates with Intraoperative Ultrasound-Guided Excision for Myxofibrosarcoma: A Single-Center Cohort Study with External Comparisons

**DOI:** 10.3390/curroncol33090538

**Published:** 2026-09-04

**Authors:** Youngkeun Lee, Sujin Lee, Sang Ah Chi, Sung Wook Seo

**Affiliations:** 1 Department of Orthopedic Surgery, Samsung Medical Center, 81 Irwon-ro, Gangnam-gu, Seoul 06351, Republic of Korea; arslyk@gmail.com; 2 Department of Digital Health, Samsung Advanced Institute for Health Sciences and Technology, Sungkyunkwan University, 115 Irwon-ro, Gangnam-gu, Seoul 06355, Republic of Korea; 2sujin2019@gmail.com; 3 Biomedical Statistics Center, Data Science Research Institute, Research Institute for Future Medicine, Samsung Medical Center, 115 Irwon-ro, Gangnam-gu, Seoul 06355, Republic of Korea; sangah22.chi@samsung.com; 4 Department of Orthopedic Surgery, School of Medicine, Sungkyunkwan University, 81 Irwon-ro, Gangnam-gu, Seoul 06351, Republic of Korea

**Keywords:** myxofibrosarcoma, intraoperative ultrasound, surgical margins, local recurrence, soft tissue sarcoma, limb salvage, matching-adjusted indirect comparison

## Abstract

**Background/Objectives**: Myxofibrosarcoma (MFS) shows infiltrative fascial extensions that complicate margin achievement and drive local recurrence. We evaluated whether intraoperative ultrasound guidance improves negative margin achievement and oncological outcomes compared with conventional excision. **Methods**: In this retrospective cohort study at a single tertiary referral center, patients with MFS underwent conventional excision (2008–2013; *n* = 15) or ultrasound-guided excision (2014–2024; *n* = 50; primary comparative analysis *n* = 47 after excluding three patients). The primary outcome was negative margin achievement; secondary outcomes were overall survival (OS), local recurrence-free survival (LRFS), and distant metastasis-free survival (DMFS). Exploratory matching-adjusted indirect comparisons (MAICs) were performed against seven published international MFS cohorts. **Results**: Overall R0 (negative) margin rates did not differ between cohorts (97.9% vs. 93.3%; *p* = 0.428). Among R0 margins, however, a wide clearance (R0-wide, ≥1 mm) was more frequent in the US-guided cohort, whereas R0-close (<1 mm) margins predominated after conventional excision (R0-wide 83.0% vs. 46.7%; odds ratio 5.38, 95% CI 1.30–23.73; *p* = 0.014). Median follow-up was 49 months; five-year OS, LRFS, and DMFS were 97.5%, 94.3%, and 83.7%. In the internal historical comparison, LRFS was directionally higher with ultrasound guidance (94.3% vs. 68.2%; HR 0.26; *p* = 0.059). In MAICs, LRFS favored the US-guided cohort in all five comparisons and OS in two of four. **Conclusions**: Intraoperative ultrasound guidance was associated with a higher rate of wide (R0-wide, ≥1 mm) negative margins and favorable local disease control in MFS; whether wider microscopic clearance itself improves local control requires prospective multicenter evaluation.

## Simple Summary

Myxofibrosarcoma is a type of soft-tissue cancer that tends to send microscopic, finger-like extensions along the tissue planes around it. These hidden extensions are difficult to see during an operation, so a surgeon may unknowingly leave cancer behind, and the tumor can regrow at the same site. In this study we examined whether using an ultrasound probe during surgery—to view the tumor edges in real time—helps surgeons remove the tumor more completely than the traditional approach based on preoperative scans alone. We compared patients operated on with and without ultrasound guidance at one hospital, and we also compared our results with those reported by other centers worldwide. Patients treated with ultrasound guidance more often had a wide, clean margin of healthy tissue around the tumor and had low rates of the cancer returning locally. These findings suggest that ultrasound guidance may improve surgery for this challenging cancer.

## 1. Introduction

Myxofibrosarcoma (MFS) is one of the most common soft tissue sarcomas in elderly patients [1], characterized by infiltrative tail-like extensions along fascial planes that render complete surgical excision inherently challenging [2,3]. Even when resection appears macroscopically adequate, occult microscopic extensions frequently result in positive or close resection margins, contributing to local recurrence rates of 14–61% across institutional series [4,5,6,7]. Because locoregional recurrence directly affects long-term survival in extremity soft tissue sarcoma [8], improving the accuracy of intraoperative margin delineation is a central objective in MFS surgery.

Preoperative MRI is the standard modality for surgical planning [9], yet it may underestimate infiltrative extensions oriented obliquely between imaging planes [2,10]. Consequently, the margin actually achieved at surgery frequently falls short of the intended margin, particularly at the distal extent of the fascial tail sign—a disparity that is often unrecognized intraoperatively. Staged excision strategies achieve local recurrence rates of approximately 5–6% [11,12], but require multiple procedures and prolonged hospitalization.

Intraoperative ultrasound has been explored as a real-time guidance tool in malignant soft tissue tumors [13]; however, available evidence derives from histologically heterogeneous cohorts, and prior studies have lacked within-institution comparators.

We therefore conducted a retrospective cohort study comparing patients who underwent conventional excision (2008–2013) with those who underwent intraoperative ultrasound-guided excision (2014–2024) at a single tertiary referral center. The primary objective was to determine whether ultrasound guidance improves the rate of negative margin achievement. Secondary objectives were to evaluate OS, LRFS, and DMFS in the US-guided cohort and to contextualize these outcomes through MAICs against seven published international MFS series.

## 2. Materials and Methods

### 2.1. Study Design and Setting

We conducted a retrospective cohort study at a single tertiary referral center. Patients who underwent surgical resection for myxofibrosarcoma of the extremities or trunk between January 2008 and December 2024 were identified from the institutional sarcoma registry. Follow-up information was updated through June 2025. The study was approved by the Institutional Review Board (IRB No. 2026-07-134), and the requirement for informed consent was waived owing to its retrospective nature. The study was conducted in accordance with the Declaration of Helsinki. As a retrospective observational cohort study, prospective trial registration was not required.

### 2.2. Patient Selection

All patients with histologically confirmed myxofibrosarcoma who underwent surgery during the study period were included. Patients were stratified into two groups based on the implementation of intraoperative ultrasound guidance: a conventional excision cohort (surgery 2008–2013, before the introduction of the intraoperative ultrasound protocol) and a US-guided excision cohort (surgery 2014–2024).

**Figure 1:**
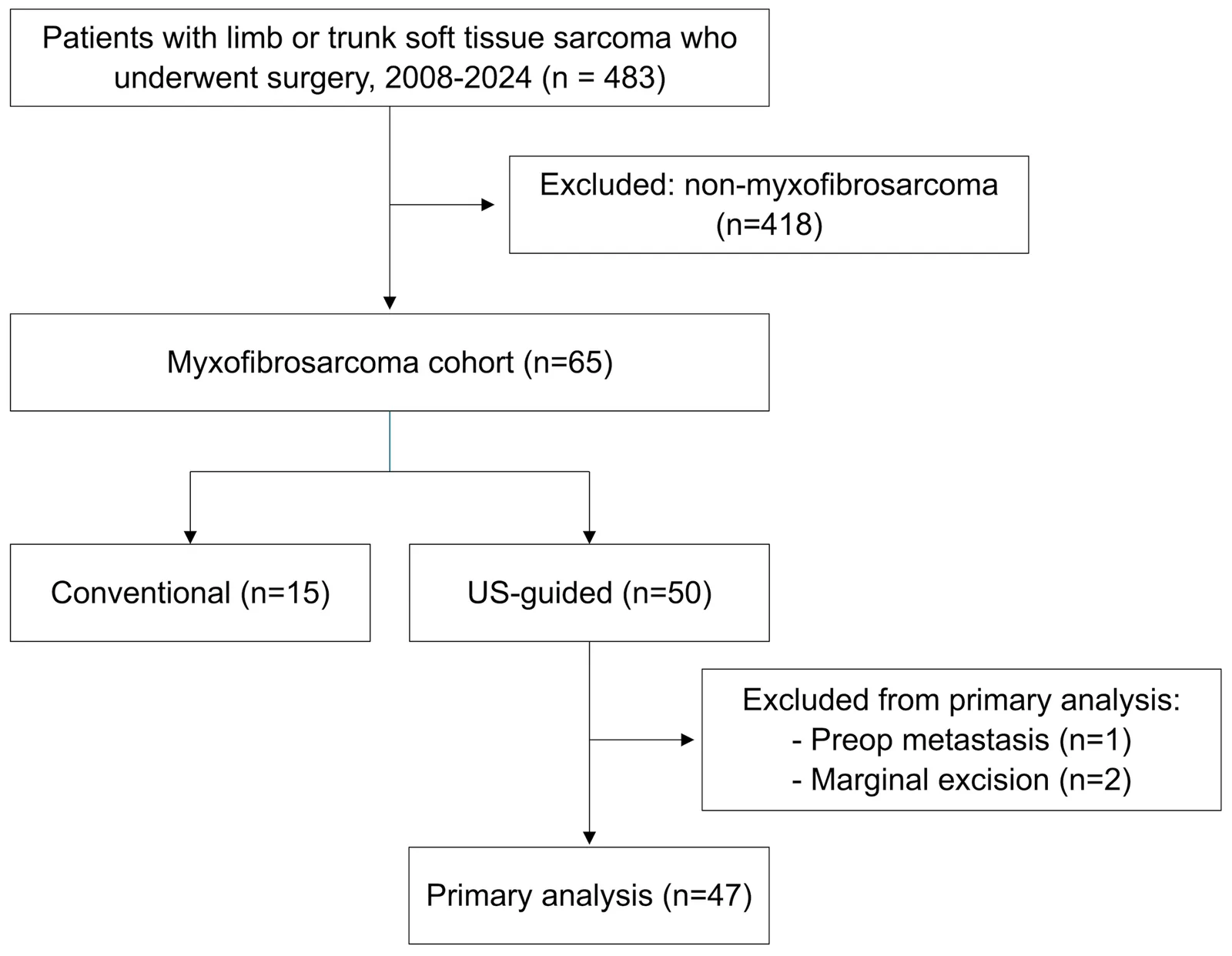
Patient selection flowchart. Of 65 patients with myxofibrosarcoma included, 15 comprised the conventional cohort (2008–2013) and 50 the US-guided cohort (2014–2024). Three patients in the US-guided cohort were excluded from primary analyses (one with preoperative metastasis; two with marginal excision) but retained for MAIC, yielding a primary analysis cohort of 47 US-guided patients.

For the primary comparative analysis, patients were excluded if they had distant metastasis at initial presentation or underwent marginal rather than wide excision due to medical comorbidities or patient refusal. These excluded patients were retained for the MAIC analyses with external cohorts, in which the appropriate subset was selected to match each comparator’s eligibility criteria (see Section 2.8). A total of 65 patients were identified: 15 in the conventional cohort and 50 in the US-guided cohort, of whom 47 were eligible for the primary comparative analysis after applying the exclusion criteria, yielding a primary analysis cohort of 62 patients (conventional, *n* = 15; US-guided, *n* = 47). The patient selection process is illustrated in Figure 1.

### 2.3. Intraoperative Ultrasound Protocol

**Figure 2:**
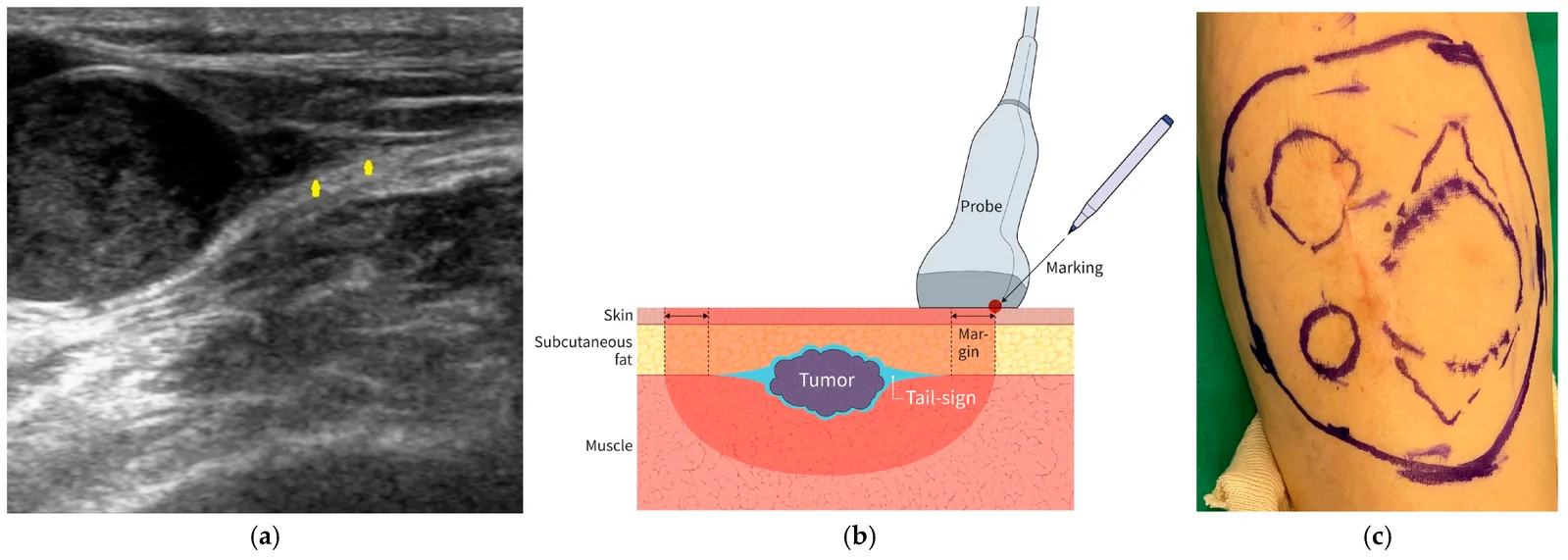
(**a**) Intraoperative ultrasound image showing delineation of the tumor margin and a tail-like extension (yellow arrows) within the fascial layer. (**b**) Schematic illustration demonstrating ultrasound-guided incision planning for myxofibrosarcoma. The incision line is marked approximately 2 cm beyond the outermost extent of the tail sign to ensure adequate surgical margins. (**c**) Intraoperative photograph showing ultrasound-guided skin marking of a myxofibrosarcoma with one main nodule and two satellite nodules. The final incision line was drawn 2 cm beyond the tail sign and satellite nodules to achieve oncologically safe margins.

In the US-guided cohort, intraoperative ultrasound was performed using a standardized protocol with a high-frequency linear transducer (L12-5, Philips iU22; Philips Healthcare, Bothell, WA, USA). One senior surgeon performed all assessments, with longitudinal, transverse, and circumferential scanning. Particular attention was paid to the tail sign—a hypoechoic linear or infiltrative area with fluid-like echogenicity, continuous with the tumor and extending along fascial planes. When a tail sign was present, its distal extent defined the reference; otherwise, the tumor’s visible boundary was used. For wide excision, a 2 cm margin was secured beyond this reference; for marginal excision, the sonographic boundary itself served as the resection margin. Patients in the conventional cohort underwent wide excision without intraoperative ultrasound guidance. Representative examples are shown in Figure 2a–c.

### 2.4. Preoperative Imaging Assessment

Preoperative MRI was reviewed for the presence of a tail sign, defined as a curvilinear T2-hyperintense fascial extension from the main tumor mass [10]; assessment was performed by the treating orthopedic oncologist, blinded to outcomes. All preoperative MRI examinations were performed on 3.0-T scanners. All patients in the conventional cohort were imaged on a single system (Achieva 3.0T; Philips Healthcare, Best, The Netherlands), whereas patients in the US-guided cohort were imaged on 3.0-T systems from two vendors (Achieva, Ingenia, and Ingenia CX, Philips Healthcare; MAGNETOM Skyra and Vida, Siemens Healthineers, Erlangen, Germany), reflecting scanner replacement at our institution over the study period.

### 2.5. Radiotherapy

Radiotherapy was not administered according to a fixed protocol. It was recommended by the institutional multidisciplinary tumor board, principally for close or microscopically involved resection margins, high FNCLCC grade, or extensive infiltrative disease. Timing, doses, and fractionation are reported in the Section 3.

### 2.6. Outcome Measures

The primary outcome was margin status at final histopathology. A margin was classified as positive (R1) when tumor reached the inked resection surface and as negative (R0) when it did not. Negative (R0) margins were further stratified by the closest clearance between tumor and the inked surface into R0-wide (≥1 mm) and R0-close (<1 mm). Final margin status was assessed by a board-certified musculoskeletal pathologist on permanent sections.

Secondary outcomes included overall survival (OS), local recurrence-free survival (LRFS), and distant metastasis-free survival (DMFS). OS was defined as the time from surgery to death from any cause. Local recurrence was defined as radiological or pathological evidence of tumor recurrence within the surgical bed or the same anatomical compartment. Distant metastasis was defined as tumor spread to a separate anatomical compartment, regional lymph nodes, or distant organs. All events were confirmed by a musculoskeletal radiologist and the treating orthopedic oncologist. Patients without events were censored at the last follow-up.

### 2.7. Follow-Up

Follow-up was conducted every three months for the first postoperative year and every six months thereafter, with clinical examination and surveillance imaging alternating between MRI of the surgical site combined with chest CT and whole-body MRI.

### 2.8. Statistical Analysis

Continuous variables were summarized as median (interquartile range, IQR) owing to small cohort sizes and non-normal distributions; categorical variables were summarized as frequency (%). Age was compared using the Wilcoxon rank-sum test, and categorical variables were compared using Fisher’s exact test. Odds ratios for margin status with 95% CIs were also obtained from Fisher’s exact test. Survival outcomes were estimated using the Kaplan–Meier method, and median follow-up was estimated by the reverse Kaplan–Meier method. Cohorts were compared using the log-rank test, and unadjusted hazard ratios (HRs) with 95% CIs were obtained from univariate Cox models. Because the conventional cohort was small (*n* = 15), these comparisons are reported as directional evidence.

MAIC [14] was performed against seven external myxofibrosarcoma cohorts, using our individual patient data together with aggregate data reconstructed from published Kaplan–Meier curves by the Guyot method [15], with coordinate extraction using Engauge Digitizer. Matching covariates (age, sex, tumor size, depth, FNCLCC grade, and radiotherapy) were selected in advance; the exact set used in each comparison depended on what each comparator reported. Margin status was not used for matching, because it is likely a consequence of ultrasound guidance rather than a baseline characteristic. For each MAIC, the US-guided cohort was restricted to patients meeting that comparator’s eligibility criteria, giving pre-matching sample sizes of 47 to 50. Weighted Cox models then provided HRs with 95% CIs for OS, LRFS, and DMFS.

Exploratory univariable Cox models for OS and LRFS were fitted for baseline variables (age, sex, tumor size, depth, FNCLCC grade, tumor location, surgical presentation, margin category, radiotherapy, and MRI tail sign). Because only five deaths and seven local recurrences occurred, the multivariable model was restricted to three covariates (treatment group, FNCLCC grade 3, and tumor size > 5 cm), and Firth’s penalized likelihood was applied when complete separation precluded standard estimation.

Data were complete for the primary analyses, except for one patient in the conventional cohort with a missing FNCLCC grade, who was excluded only from grade-specific comparisons. A *p*-value of <0.05 was considered statistically significant. All analyses were performed in R (version 4.5.0).

## 3. Results

Baseline characteristics are summarized in Table 1. The conventional cohort comprised 15 patients (median age 58.1 years; 8 [53.3%] female), and the US-guided cohort comprised 47 patients (median age 61.1 years; 18 [38.3%] female). The two cohorts were comparable in age, sex, tumor depth, FNCLCC grade, MRI tail sign prevalence, and surgical presentation (all *p* > 0.25; Table 1). Among the 28 patients who presented after an unplanned excision performed elsewhere, residual tumor was present in the re-excision specimen in 26 (92.9%; 5 of 5 in the conventional cohort and 21 of 23 in the US-guided cohort; Table 1). Radiotherapy was administered in 26 of 62 patients (41.9%): postoperatively in 25 and preoperatively in 1. The median total dose was 60 Gy (range 24–66 Gy) delivered over a median of 30 fractions (range 8–33); dose and fractionation details were unavailable for 3 patients treated at other institutions or in the early study period.

By the R0/R1 classification, negative (R0) margins were achieved in 14 of 15 conventional patients (93.3%) and 46 of 47 US-guided patients (97.9%); this difference was not statistically significant (*p* = 0.428, Fisher’s exact test), and positive (R1) margins were uncommon in both cohorts (1 of 15 vs. 1 of 47). Among R0 margins, the clearance width differed: an R0-wide (≥1 mm) margin was achieved in 39 of 47 US-guided patients (83.0%) versus 7 of 15 conventional patients (46.7%), with R0-close (<1 mm) margins making up the remainder (odds ratio for an R0-wide versus R0-close or positive margin 5.38, 95% CI 1.30–23.73; *p* = 0.014; Table 2).

### 3.1. Oncological Outcomes

**Figure 3:**
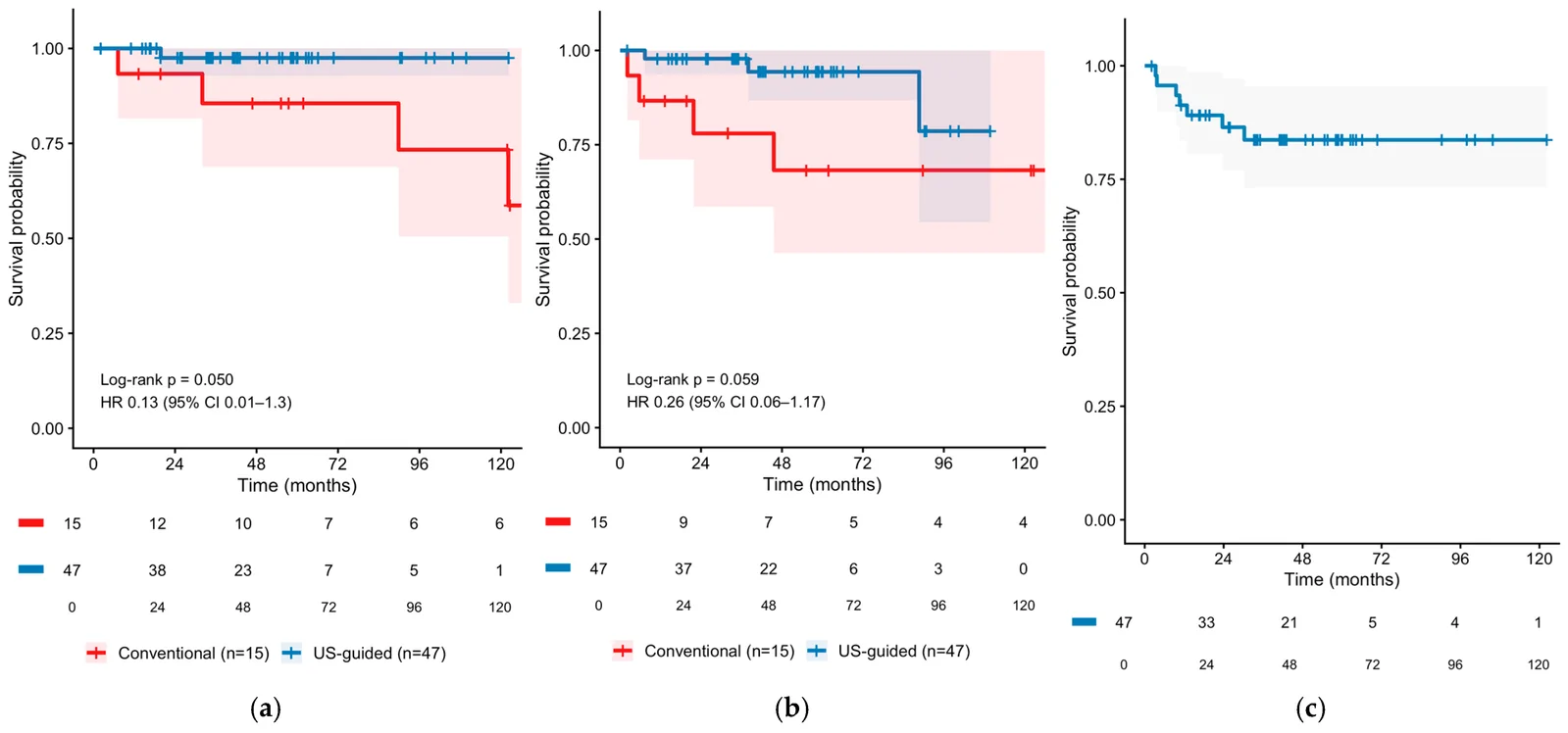
Kaplan–Meier survival curves. (**a**) Overall survival and (**b**) local recurrence-free survival comparing the conventional (*n* = 15) and US-guided (*n* = 47) cohorts. (**c**) Distant metastasis-free survival in the US-guided cohort. Shaded areas represent 95% confidence intervals; tick marks indicate censored observations; number-at-risk tables are shown below each curve. HR, hazard ratio.

During a median follow-up of 49 months (IQR 26–61), death occurred in 1 of 47 patients (2.1%), local recurrence in 3 (6.4%), and distant metastasis in 7 (14.9%). The three-year and five-year OS rates were both 97.5% (95% CI 92.8–100). LRFS rates at three and five years were 97.8% (95% CI 93.7–100) and 94.3% (95% CI 86.8–100), respectively. DMFS was 83.7% (95% CI 73.2–95.6) at both three and five years (Figure 3c). LRFS did not differ significantly by tumor depth (superficial vs. deep-seated: 95.0% vs. 94.7%; *p* = 0.860) or surgical presentation (primary resection vs. prior unplanned excision: 100% vs. 95.7%; *p* = 0.404). Local recurrence was observed in 1 of 5 trunk tumors (20.0%) and 2 of 42 extremity tumors (4.8%); formal comparison was not performed owing to the small trunk subgroup.

Compared with the conventional excision cohort (*n* = 15), the US-guided cohort demonstrated directionally favorable OS and LRFS. Death occurred in 4 of 15 conventional excision patients (26.7%) versus 1 of 47 US-guided patients (2.1%). The five-year OS was 85.6% in the conventional cohort and 97.5% in the US-guided cohort (hazard ratio [HR] 0.13, 95% CI 0.01–1.30; log-rank *p* = 0.050; Figure 3a). Local recurrence occurred in 4 of 15 conventional excision patients (26.7%) versus 3 of 47 US-guided patients (6.4%). The five-year LRFS was 68.2% versus 94.3% (HR 0.26, 95% CI 0.06–1.17; log-rank *p* = 0.059; Figure 3b). Statistical power for these comparisons is limited owing to the small conventional excision cohort (*n* = 15); results should be interpreted as directional evidence.

In exploratory univariable Cox models, no baseline variable was significantly associated with OS or LRFS (Table S4). In the multivariable model for LRFS (treatment group, FNCLCC grade 3, and tumor size > 5 cm), the direction of the group effect was preserved (HR 0.40, 95% CI 0.07–2.12). A multivariable model for OS was not estimable because all five deaths occurred in patients with grade 3 tumors. Given the small number of events, these regression analyses are exploratory.

### 3.2. External Comparison

Exploratory MAICs were performed against seven published MFS cohorts [1,5,6,7,16,17,18]; the pre-weighting sample size of the US-guided cohort ranged from 47 to 50 depending on each comparator’s eligibility criteria. Comparative analyses were feasible for four OS, five LRFS, and one DMFS comparison.

**Figure 4:**
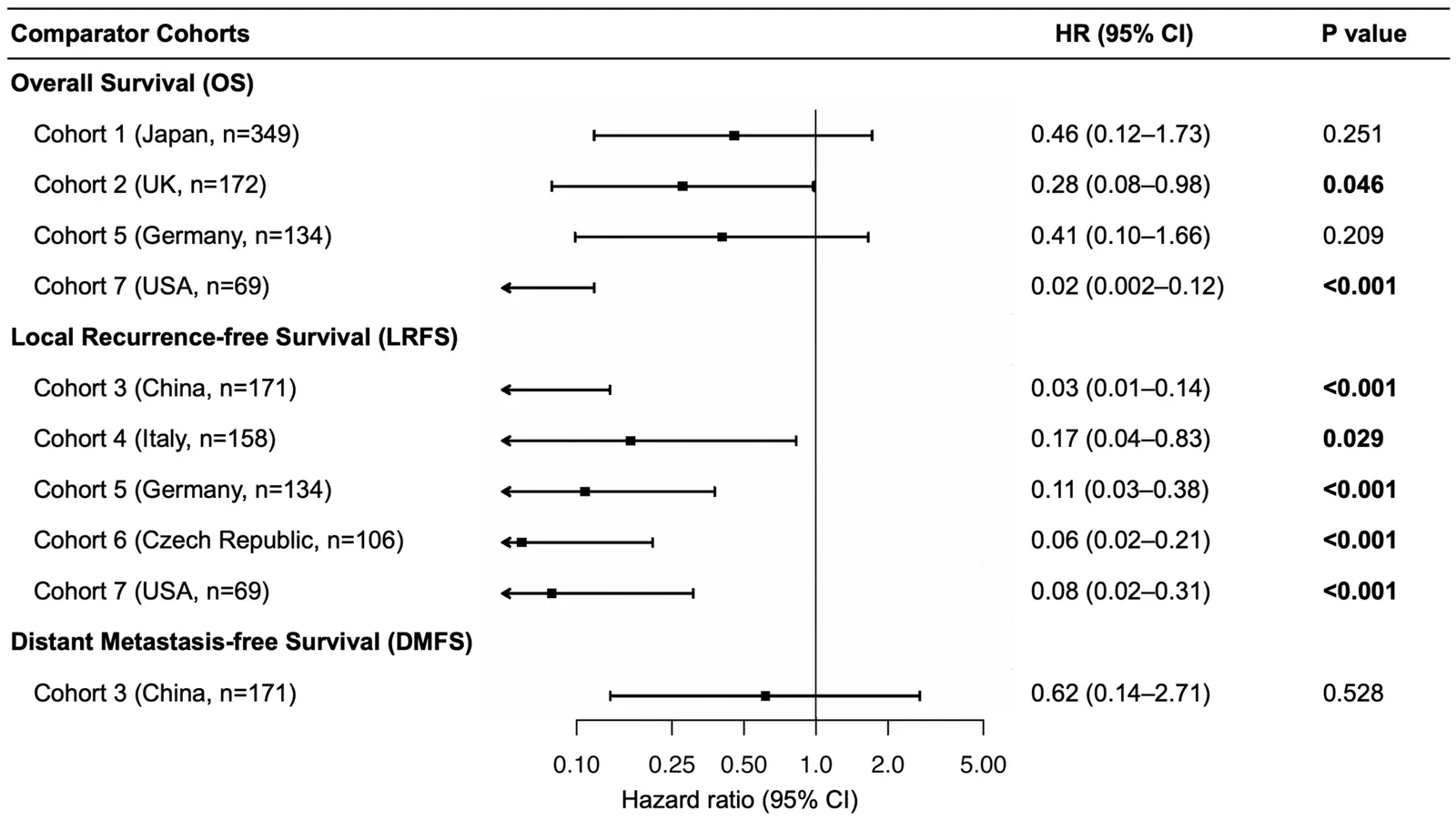
Matching-adjusted indirect comparison of OS, LRFS, and DMFS between the US-guided cohort and seven external MFS cohorts. Each comparator cohort was reweighted by MAIC to balance baseline covariates against the US-guided index cohort. Horizontal lines represent 95% CIs; filled squares denote point estimates. An HR < 1.0 favors the US-guided cohort. Arrows indicate that the 95% CI extends beyond the displayed axis range. Bold *p*-values indicate statistical significance (*p* < 0.05). Vertical solid line indicates HR = 1.0 (no effect). Comparator cohorts are as follows: Cohort 1 [1]; Cohort 2 [16]; Cohort 3 [6]; Cohort 4 [5]; Cohort 5 [17]; Cohort 6 [18]; Cohort 7 [7].

All four OS hazard ratios favored the US-guided cohort, with statistical significance achieved in two comparisons (HR 0.02, 95% CI 0.002–0.12; *p* < 0.001 vs. Cohort 7; HR 0.28, 95% CI 0.08–0.98; *p* = 0.046 vs. Cohort 2). For LRFS, effect estimates consistently favored the US-guided cohort across all five comparisons (HR range 0.03–0.17), each meeting statistical significance (*p* range < 0.001 to 0.029). For DMFS, only one comparison was feasible and no significant difference was observed (HR 0.62, 95% CI 0.14–2.71; *p* = 0.530). These results are summarized in Figure 4. Additional cohort summaries, baseline characteristics, matching variables, and MAIC weighting outputs are provided in Tables S1–S3.

## 4. Discussion

In this cohort study of patients undergoing surgery for myxofibrosarcoma, intraoperative ultrasound guidance was associated with a higher rate of wide (R0-wide, ≥1 mm) negative margins and with favorable local recurrence outcomes, although whether the former mechanistically drives the latter cannot be established from these data.

The difficulty of achieving negative margins in MFS stems partly from a mismatch between preoperative imaging and intraoperative reality. MRI provides accurate macroscopic delineation but may underestimate fascial extensions oriented obliquely between imaging planes; as a result, the achieved margin frequently falls short of what was intended, particularly at the distal extent of the fascial tail. Even a microscopically negative margin in MFS is associated with a relatively high local recurrence rate compared with other soft tissue sarcoma subtypes [19], and a positive (R1) margin is an independent predictor of reduced LRFS [20], underscoring the value of maximizing margin quality; in extremity and trunk STS, planned collaboration with reconstructive surgery has likewise been used to achieve adequate oncological margins in resections that would otherwise be compromised [21]. Real-time sonographic visualization allows the surgeon to identify and respond to these extensions at the time of resection, which may explain the more frequent achievement of R0-wide (≥1 mm) margins in the US-guided cohort compared with conventional excision (83.0% vs. 46.7%; OR 5.38, 95% CI 1.30–23.73); intraoperative ultrasound has similarly been used to localize and confirm complete excision of soft tissue tumors [22], although current reviews conclude that ultrasound and other intraoperative margin-assessment techniques still require prospective validation [23]. Notably, this difference was confined to the clearance width (R0-wide versus R0-close); overall R0 rates were high and did not differ significantly between cohorts. The prognostic weight of a sub-millimeter (close) margin is itself contested: some series report that close margins independently increase local recurrence risk and that a minimal clearance of at least 1 mm improves local control [24,25], whereas others find that a negative but <1 mm (“R + 1 mm”) margin may be oncologically adequate, with local recurrence rates approaching those of wider negative margins [26]. Our observation of more frequent R0-wide (≥1 mm) margins under ultrasound guidance should therefore be regarded as a hypothesis-generating signal whose clinical significance requires dedicated prospective evaluation, rather than as established evidence that the sub-millimeter distinction alters outcome. Staged excision with vacuum-assisted closure has achieved local recurrence rates of 5–6% in selected series [11,12], comparable to the present cohort. However, staged approaches require multiple procedures and prolonged hospitalization. Whether a single-stage ultrasound-guided approach can reliably achieve equivalent margin quality requires prospective comparison.

Patients treated before the adoption of intraoperative ultrasound (2008–2013) provide a reference group that shares the same surgical team, pathology assessment, and referral patterns. Local recurrence occurred in 26.7% of the conventional cohort versus 6.4% of the US-guided cohort, and OS was similarly directionally favorable. The local recurrence rate in our US-guided cohort (6.4%) compares favorably with contemporary MFS series, in which five-year local recurrence is typically around 10–12% even with routine perioperative radiotherapy [20,27,28]. That said, the conventional cohort is small (*n* = 15), and the two cohorts span 17 years, so an era effect cannot be excluded. Over the study period, MRI platforms were replaced (although all examinations were performed at 3.0 T) and radiotherapy techniques evolved. The infiltrative growth pattern of MFS also became more widely appreciated, plausibly leading surgeons to plan wider margins in the recent era, and the experience of the surgical team and the degree of multidisciplinary integration increased, in line with the current emphasis on multidisciplinary management in sarcoma referral centers [29]. Any of these changes may have contributed to the observed differences. The internal comparison is therefore best interpreted as directional rather than causal evidence.

LRFS did not differ significantly between primary resection and prior unplanned excision subgroups, despite prior unplanned surgery being an established predictor of local recurrence in MFS and soft tissue sarcoma more broadly [7,30,31,32,33]. Residual tumor was present in the re-excision specimen in 26 of 28 patients (92.9%) who presented after an unplanned excision. During re-excision, intraoperative ultrasound was used in the same manner as for primary tumors: the previous surgical bed, including scar tissue, satellite nodules, and any tail-like fascial extension, was delineated sonographically, and the resection margin was set beyond this boundary. Real-time delineation of residual tumor extent may partly explain this observation, though subgroup sizes preclude formal conclusions. Similarly, LRFS did not differ by tumor depth, suggesting sonographic guidance provides useful information in both superficial and deep-seated tumors.

LRFS consistently favored the US-guided cohort across all five external MAICs, and OS significantly favored the US-guided cohort in two of four comparisons, with all four hazard ratios directionally consistent. No difference in DMFS was observed, consistent with the expectation that local surgical technique primarily influences locoregional disease control. This directional consistency across geographically and temporally diverse cohorts lends additional context to the within-institution findings.

Each MAIC reweights the index cohort to match a different external reference population [14], such that the comparison populations differ across analyses. Unmeasured prognostic factors—including race and ethnicity, comorbidities, and radiotherapy technique—cannot be adjusted for using aggregate-data MAIC. These comparisons should therefore be understood as contextual evidence, not formal controlled comparisons.

There are several limitations. First, regarding the technique itself: all intraoperative assessments were performed by a single senior surgeon with no evaluation of inter-operator reproducibility, and findings were not prospectively recorded as discrete variables, precluding histopathological correlation between sonographic and pathological tumor extent. Moreover, ultrasound resolves only macroscopic tumor boundaries and cannot detect microscopic infiltration beyond the sonographic margin; a sonographically clear margin therefore does not guarantee the absence of residual microscopic disease. Second, regarding the within-institution comparison: the small conventional cohort limits statistical power and era-related confounders cannot be excluded. Third, regarding external comparisons: each MAIC applies to a different reweighted population, unmeasured confounders cannot be adjusted for, and individual-level data were unavailable. Fourth, although intraoperative ultrasound may allow the resection margin to be tailored more precisely than macroscopic MRI delineation alone, we did not compare functional outcomes against a strategy of empirically widening the margin on the basis of MRI; whether the more conformal margin translates into a measurable functional benefit—as opposed to merely reducing the volume of tissue removed—was not assessed and warrants prospective, function-focused evaluation.

## 5. Conclusions

Intraoperative ultrasound guidance was associated with higher rates of wide (R0-wide, ≥1 mm) negative margins in patients undergoing surgery for myxofibrosarcoma, and oncological outcomes in the US-guided cohort were favorable. These findings do not establish that the wider microscopic clearance itself improves local control, and era-related factors may have contributed to the differences observed in the internal comparison. Prospective multicenter studies with standardized ultrasound protocols, formal inter-operator assessment, and histopathological correlation are needed to establish the reproducibility and generalizability of this approach.

## Figures and Tables

**Table 1:** Baseline characteristics of the conventional and US-guided excision cohorts.

Characteristic	Overall (*n* = 62)	Conventional (*n* = 15)	US-Guided (*n* = 47)	*p* Value ^†^
Age at surgery, years	61.9 (54.4–69.9)	58.1 (52.0–69.1)	61.1 (57.1–70.1)	0.260
Sex				0.373
Male	36 (58.1%)	7 (46.7%)	29 (61.7%)	
Female	26 (41.9%)	8 (53.3%)	18 (38.3%)	
Location				0.901
Lower	40 (64.5%)	11 (73.3%)	29 (61.7%)	
Trunk	6 (9.7%)	1 (6.7%)	5 (10.6%)	
Upper	16 (25.8%)	3 (20.0%)	13 (27.7%)	
Tumor size				0.755
≤5 cm	41 (66.1%)	9 (60.0%)	32 (68.1%)	
>5 cm	21 (33.9%)	6 (40.0%)	15 (31.9%)	
Depth				>0.999
Superficial	35 (56.5%)	8 (53.3%)	27 (57.4%)	
Deep	27 (43.5%)	7 (46.7%)	20 (42.6%)	
FNCLCC grade				0.670
Low-grade, grade 1	9 (14.8%)	1 (7.1%)	8 (17.0%)	
High-grade, grade 2–3	52 (85.2%)	13 (92.9%)	39 (83.0%)	
MRI tail sign				0.322
Absent	15 (24.2%)	2 (13.3%)	13 (27.7%)	
Present	47 (75.8%)	13 (86.7%)	34 (72.3%)	
Surgical presentation				0.347
Primary	21 (33.9%)	5 (33.3%)	16 (34.0%)	
Prior unplanned excision	28 (45.2%)	5 (33.3%)	23 (48.9%)	
Residual tumor in re-excision specimen ^‡^	26/28 (92.9)	5/5 (100.0)	21/23 (91.3)	
Recurrent	13 (21.0%)	5 (33.3%)	8 (17.0%)	
Radiotherapy	26 (41.9%)	9 (60.0%)	17 (36.2%)	0.137

^†^ Conventional vs. US-guided comparison; Fisher’s exact test; Wilcoxon rank-sum test for age. Values are presented as median (IQR) for continuous variables and *n* (%) for categorical variables. FNCLCC, Fédération Nationale des Centres de Lutte Contre le Cancer; MRI, magnetic resonance imaging. ^‡^ Denominators are patients with a prior unplanned excision.

**Table 2:** Margin status comparison between the conventional and US-guided cohorts.

Characteristic	Overall (*n* = 62)	Conventional (*n* = 15)	US-Guided (*n* = 47)	*p* Value ^†^
Margin status				0.009
R0-wide (≥1 mm)	46 (74.2%)	7 (46.7%)	39 (83.0%)	
R0-close (<1 mm)	14 (22.6%)	7 (46.7%)	7 (14.9%)	
Positive (R1)	2 (3.2%)	1 (6.7%)	1 (2.1%)	
R0-wide margin OR (95% CI) ^‡^		Reference	5.38 (1.30–23.73)	**0.014**
R0 resection (R0-wide + R0-close)	60 (96.8%)	14 (93.3%)	46 (97.9%)	0.428

^†^ Fisher’s exact test (Monte Carlo simulation, B = 10,000 for 3-category comparison). Bold value indicate statistically significant results (*p* < 0.05). ^‡^ Odds ratio for achieving an R0-wide (≥1 mm) margin (R0-wide vs. R0-close + positive); estimated by Fisher’s exact test.

## Data Availability

The de-identified individual patient data supporting the findings of this study, together with a variable codebook, are openly available in Mendeley Data at https://doi.org/10.17632/tnhggg84br.1. The aggregate data used for the matching-adjusted indirect comparisons were reconstructed from Kaplan–Meier curves published in the cited external studies and are therefore not redistributed here.

## Supplementary Materials

The following supporting information can be downloaded at https://www.mdpi.com/article/10.3390/curroncol33090538/s1, Table S1: Summary of study cohorts and matching covariates used in each MAIC comparison; Table S2: Baseline characteristics of our study cohort and external comparator cohorts before MAIC weighting; Table S3: Baseline characteristics of our study cohort after MAIC weighting vs. external comparator cohorts; Table S4: Exploratory univariable and multivariable Cox regression analyses for overall survival and local recurrence-free survival. References [1,5,6,7,16,17,18] are cited in the Supplementary Materials.

## Abbreviations

The following abbreviations are used in this manuscript:

MFS	Myxofibrosarcoma
US	Ultrasound
MRI	Magnetic resonance imaging
CT	Computed tomography
OS	Overall survival
LRFS	Local recurrence-free survival
DMFS	Distant metastasis-free survival
MAIC	Matching-adjusted indirect comparison
HR	Hazard ratio
CI	Confidence interval
IQR	Interquartile range
FNCLCC	Fédération Nationale des Centres de Lutte Contre le Cancer
IRB	Institutional Review Board
R0	Microscopically negative resection margin (no tumor at the inked surface)
R1	Microscopically positive resection margin (tumor at the inked surface)
