# Supplementary material for: Higher Negative Margin Rates with Intraoperative Ultrasound-Guided Excision for Myxofibrosarcoma: A Single-Center Cohort Study with External Comparisons
Source: Curr Oncol. 2026 Sep 4;33(9):538. doi: 10.3390/curroncol33090538 (PMC13605930; doi:10.3390/curroncol33090538)
Supplement: Supplementary file 1 [file curroncol-33-00538-s001.zip › Supplementary Table S1.pdf]

Supplementary Table S1. Summary of study cohorts and matching covariates used in each MAIC comparison

Cohort	Country	Study (Year, Journal)	Institution / Data Source	Period	No. of patients	Surgical selection	Metastasis at Diagnosis	OS Definition	Outcome compared (5yr)	Matching covariates	ESS
Our study cohort (IPD)	—	—	Single tertiary referral center	2014–2025	50	Any surgery (Wide & Marginal)	Included	All-cause death	—	—	—
Comparator cohort 1 (AGD)	Japan	Kamio et al., Ann Surg Oncol 2023 [1]	Bone & Soft Tissue Tumor Registry, Japan	2006–2015	349	Any surgery (Wide & Marginal)	Excluded	All-cause death	OS	depth<br>grade<br>RT	26.7
Comparator cohort 2 (AGD)	UK	Dewan et al., Sarcoma 2012 [16]	Royal Orthopaedic Hospital, Birmingham	1993–2007	172	All patients (incl. non-surgical)	Included	All-cause death	OS	age<br>size<br>depth<br>grade	32.3
Comparator cohort 3 (AGD)	China	Liu et al., Ann Transl Med 2021 [6]	Cancer Hospital, Chinese Academy of Medical Sciences	1999–2018	171	Any surgery (Wide & Marginal)	Excluded	Disease-specific (cancer death)	LRFS_3yr<br>DMFS_3yr	grade	13.2
Comparator cohort 4 (AGD)	Italy	Sanfilippo et al., Ann Surg Oncol 2011 [5]	Istituto Nazionale Tumori, Milan	1994–2009	158	Wide excision only	Excluded	All-cause death	LRFS	age<br>sex<br>size<br>depth<br>grade<br>RT	27.7
Comparator cohort 5 (AGD)	Germany	Pogkas et al., The Oncologist 2024 [17]	Helios Klinikum Berlin Buch	1998-2020	134	All patients (incl. non-surgical)	Excluded	All-cause death	OS<br>LRFS	age<br>sex<br>size<br>grade<br>RT	20.8

Comparator cohort 6 (AGD)	Czech Republic	Tomáš et al., Sci Rep 2024 [18]	Masaryk University / St. Anne's Univ Hospital	2010–2022	106	Any surgery (Wide & Marginal)	Included	Disease-specific (cancer death)	LRFS	depth grade RT	32.7
Comparator cohort 7 (AGD)	USA	Look Hong et al., Ann Surg Oncol 2013 [7]	Massachusetts General Hospital	1995–2010	69	Wide excision only	Included	All-cause death	OS LRFS	age sex size grade RT	17.8

Abbreviations: MAIC = Matching-Adjusted Indirect Comparison; IPD = Individual Patient Data; AGD = Aggregated Data; OS = Overall Survival; LRFS = Local Recurrence Free Survival; DMFS = Distant Metastasis Free Survival; ESS = Effective Sample Size; RT = Radiation Therapy;
