# Supplementary material for: Higher Negative Margin Rates with Intraoperative Ultrasound-Guided Excision for Myxofibrosarcoma: A Single-Center Cohort Study with External Comparisons
Source: Curr Oncol. 2026 Sep 4;33(9):538. doi: 10.3390/curroncol33090538 (PMC13605930; doi:10.3390/curroncol33090538)
Supplement: Supplementary file 1 [file curroncol-33-00538-s001.zip › Supplementary Table S2.pdf]

Supplementary Table S2. Baseline characteristics of our study cohort and external comparator cohorts before MAIC weighting

Variable	Our study cohort	Cohort 1 [1]	Cohort 2 [16]	Cohort 3 [6]	Cohort 4 [5]	Cohort 5 [17]	Cohort 6 [18]	Cohort 7 [7]
Sample size (n)	50	349	172	171	158	134	106	69
Age (years), median (range)	62.5 (41–93)	71.0 (22–94)†	68.0 (15–93)	53.0 (16–80) †	64.0 (IQR 54–72)	NR (mean 65 ± 14)	NR (49.1% ≥ 65 yrs)†	62.0 (19–94)
Female (%)	36.0	43.3†	NR	35.1†	43.7	45.5	49.1†	44.9
Tumor size > 5 cm (%)	36.0	67.3†	mean 8.4 (range 0.3–31)	48.0†	57.6 (≥ 5 cm)	70.4†	69.8†	median 6.0 (range 1–27)
Deep (%)	44.0	66.5	54.1	NR	56.3	NR	61.3	NR
High grade (%)	84.0	65.3	86.6	22.8	77.8	91.0	67.9	87.0
Radiotherapy (%)	36.0	18.6	NR†	35.1†	51.3	63.4	45.3	76.8

High grade was defined as FNCLCC grade 2 or 3, and low grade as grade 1.

Proportions were calculated among patients with available data (missing data: Cohort 6, tumor size 14%, FNCLCC grade 0.7%).

† Variables available in the external study but excluded from MAIC matching because weight estimation did not converge.

Abbreviations: MAIC = Matching-Adjusted Indirect Comparison; FNCLCC = Fédération Nationale des Centres de Lutte Contre le Cancer; NR, not reported.
