# Supplementary material for: Higher Negative Margin Rates with Intraoperative Ultrasound-Guided Excision for Myxofibrosarcoma: A Single-Center Cohort Study with External Comparisons
Source: Curr Oncol. 2026 Sep 4;33(9):538. doi: 10.3390/curroncol33090538 (PMC13605930; doi:10.3390/curroncol33090538)
Supplement: Supplementary file 1 [file curroncol-33-00538-s001.zip › Supplementary Table S3.pdf]

Supplementary Table S3. Baseline characteristics of our study cohort after MAIC weighting vs. external comparator cohorts

A. Baseline characteristics after MAIC weighting vs. Cohort 1

Variable	Our study cohort (Before weighting)	Our study cohort (After weighting)	Cohort 1 [1]
Sample size (n)	49	26.7	349
Deep (%)	42.9	66.5	66.5
High grade (%) (FNCLCC 2 + 3)	83.7	65.3	65.3
Radiotherapy (%)	36.7	18.6	18.6

B. Baseline characteristics after MAIC weighting vs. Cohort 2

Variable	Our study cohort (Before weighting)	Our study cohort (After weighting)	Cohort 2 [16]
Sample size (n)	50	32.3	172
Age (years), median (range)	62.5 (41–93)	68.3 (41–93)	68.0 (15–93)
Tumor size (cm), mean (range)	5.4 (1–30)	8.4 (1–30)	8.4 (0.3–31)
Deep (%)	44.0	54.1	54.1
High grade (%) (FNCLCC 2 + 3)	84.0	86.6	86.6

C. Baseline characteristics after MAIC weighting vs. Cohort 3

Variable	Our study cohort (Before weighting)	Our study cohort (After weighting)	Cohort 3 [6]
Sample size (n)	49	13.2	171
High grade (%) (FNCLCC 2 + 3)	83.7	22.8	22.8

D. Baseline characteristics after MAIC weighting vs. Cohort 4

Variable	Our study cohort (Before weighting)	Our study cohort (After weighting)	Cohort 4 [5]
Sample size (n)	47	27.7	158
Age (years), median (range)	61.1 (41–91)	64.4 (41–91)	64.0 (IQR 54–72)
Female (%)	38.3	43.7	43.7
Tumor size ≥ 5 cm (%)	31.9	57.6	57.6
Deep (%)	42.6	56.3	56.3
High grade (%) (FNCLCC 2 + 3)	83.0	77.8	77.8
Radiotherapy (%)	36.2	51.3	51.3

E. Baseline characteristics after MAIC weighting vs. Cohort 5

Variable	Our study cohort (Before weighting)	Our study cohort (After weighting)	Cohort 5 [17]
Sample size (n)	49	20.8	134
Age (years), mean (± sd)	64.1 (± 12)	65.0 (± 12)	65.0 (± 14)
Female (%)	36.7	45.5	45.5
Tumor size > 5 cm (%)	34.4	70.4	70.4
High grade (%) (FNCLCC 2 + 3)	83.7	91.0	91.0
Radiotherapy (%)	36.7	63.4	63.4

F. Baseline characteristics after MAIC weighting vs. Cohort 6

Variable	Our study cohort (Before weighting)	Our study cohort (After weighting)	Cohort 6 [18]
Sample size (n)	50	32.7	106
Deep (%)	44.0	61.3	61.3
High grade (%) (FNCLCC 2 + 3)	84.0	67.9	67.9
Radiotherapy (%)	36.0	45.3	45.3

G. Baseline characteristics after MAIC weighting vs. Cohort 7

Variable	Our study cohort (Before weighting)	Our study cohort (After weighting)	Cohort 7 [7]
Sample size (n)	48	17.8	69
Age (years), median (range)	61.5 (41–91)	62.7 (41–91)	62.0 (19–94)
Female (%)	37.5	44.9	44.9
Tumor size (cm), median (range)	4.0 (1–30)	6.1 (1–30)	6.0 (1–27)
High grade (%) (FNCLCC 2 + 3)	83.3	87.0	87.0
Radiotherapy (%)	35.4	76.8	76.8
