# Supplementary material for: Higher Negative Margin Rates with Intraoperative Ultrasound-Guided Excision for Myxofibrosarcoma: A Single-Center Cohort Study with External Comparisons
Source: Curr Oncol. 2026 Sep 4;33(9):538. doi: 10.3390/curroncol33090538 (PMC13605930; doi:10.3390/curroncol33090538)
Supplement: Supplementary file 1 [file curroncol-33-00538-s001.zip › Supplementary Table S4.pdf]

Supplementary Table S4. Exploratory univariable and multivariable Cox regression analyses for overall survival and local recurrence-free survival.

	OS		LRFS	
**Variable**	**HR (95% CI)**	**p value**	**HR (95% CI)**	**p value**
**Univariable analyses**				
US-guided excision (vs. conventional)	0.13 (0.01–1.30)	0.083	0.26 (0.06–1.17)	0.078
Age (per year)	0.99 (0.92–1.06)	0.714	0.99 (0.93–1.06)	0.821
Female (vs. male)	1.62 (0.26–9.91)	0.603	0.91 (0.20–4.13)	0.901
Tumor size >5 cm (vs. ≤5 cm)	5.88 (0.88–39.13)	0.067	2.61 (0.52–13.12)	0.245
Deep-seated (vs. superficial)	2.43 (0.40–14.87)	0.337	1.35 (0.30–6.16)	0.695
FNCLCC grade 3 (vs. grade 1–2)[b]	NE[a]	—	3.58 (0.61–20.84)	0.157
Trunk location (vs. extremity)	NE[a]	—	2.09 (0.25–17.74)	0.500
Prior unplanned excision (vs. others)	0.51 (0.08–3.04)	0.456	0.85 (0.19–3.84)	0.837
R0-wide margin (vs. R0-close/R1)	0.54 (0.09–3.25)	0.499	1.93 (0.23–16.28)	0.544
Radiotherapy	2.69 (0.44–16.50)	0.284	0.31 (0.04–2.56)	0.274
MRI tail sign present	0.87 (0.09–8.91)	0.907	NE[a]	—
**Multivariable analysis (LRFS)[c]**				
US-guided excision (vs. conventional)	—	—	0.40 (0.07–2.12)	0.281
FNCLCC grade 3 (vs. grade 1–2)[b]	—	—	4.11 (0.63–27.01)	0.141
Tumor size >5 cm (vs. ≤5 cm)	—	—	5.30 (0.78–35.85)	0.087

Exploratory Cox proportional hazards analyses in the primary cohort (n = 62; 5 deaths, 7 local recurrences). Dashes indicate values not reported because the estimate was not obtainable.

HR, hazard ratio; CI, confidence interval; LRFS, local recurrence-free survival; NE, not estimable; OS, overall survival.

[a] Not estimable owing to complete separation: all five deaths occurred in patients with grade 3 tumors, no deaths occurred in trunk tumors, and all seven local recurrences occurred in patients with an MRI tail sign.

[b] One patient with a missing FNCLCC grade was excluded from analyses involving grade (n = 61).

[c] A multivariable model for OS was not estimable owing to complete separation by FNCLCC grade; the multivariable model for LRFS included treatment group, FNCLCC grade 3, and tumor size >5 cm.
